# Sublingual or Subcutaneous immunotherapy for Seasonal Allergic Rhinitis (AR): an indirect analysis of efficacy, safety and cost

**DOI:** 10.1186/1710-1492-10-S1-A11

**Published:** 2014-03-03

**Authors:** George Dranitsaris, Anne K Ellis

**Affiliations:** 1 Augmentium Pharma Consulting, Toronto, M4K 3H4, ON, Canada; 2 Division of Allergy, Department of Medicine, Queen’s University, Kingston, K7L 3N6, ON, Canada

## Background

The current standard of preventive care for poorly controlled seasonal AR symptoms is subcutaneous immunotherapy (SCIT) with allergen extracts, administered in a physician’s office. As an alternative to SC administration, sublingual immunotherapy (SLIT) is now an option for patients. Oralair™ and Grazax™ are two SLIT agents currently available in many countries. However, head to head comparative data between the three options are not available. In this study, an indirect comparison on efficacy, safety and cost was undertaken between Oralair™, Grazax™ and SCIT.

## Methods

A systematic review of major databases was conducted from January 1980 to December 2012 for double blind placebo controlled randomized trials evaluating Oralair™, Grazax™ or SCIT in patients with grass-induced seasonal AR. Using placebo as the common control, an indirect statistical comparison between treatments was performed using meta regression analysis with active drug as the primary independent variable. Other variables considered in the regression model included year of study publication, geographic region where the trial was conducted, trial duration, duration of immunotherapy, number of asthmatic patients enrolled in the trial, number of allergens and patient type (adults vs. children). A cost comparison, which included costs for drug therapy, pharmacy fees, physician visits and indirect costs (i.e. patient travel and lost productivity) was also undertaken.

## Results

Overall, 20 placebo-controlled trials met the inclusion criteria for indirect analysis. Keeping in mind the caveats associated with comparisons across clinical trials, the indirect analysis suggested a possibility for improved efficacy with Oralair™ over SCIT (standardized mean difference [SMD] in AR symptom control = - 0.21; p = 0.007) and Grazax™ (SMD = - 0.18; p = 0.018). In addition, the meta regression analysis did not identify significant differences in the risk of discontinuation due adverse events between the three therapies. Oralair™ was also associated with cost savings against year round SCIT ($2,471), seasonal SCIT ($948) and Grazax™ ($1,168) during the first year of therapy.

## Conclusions

Through an indirect comparison of placebo controlled trials, the evaluation suggested that Oralair™ has at least non-inferior efficacy and comparable safety against SCIT and Grazax™ at a lower annual cost.

